# Case Report: Successful trastuzumab deruxtecan rechallenge after disease progression in a pretreated *HER2*-mutated lung adenocarcinoma patient

**DOI:** 10.3389/fonc.2026.1832498

**Published:** 2026-08-10

**Authors:** Wenji Yan, Chang Liu, Tao Li, Yi Hu, Weihong Zhao

**Affiliations:** 1 Senior Department of Medical Oncology, Chinese People's Liberation Army (PLA) General Hospital, Beijing, China; 2 School of Medicine, Nankai University, Tianjin, China

**Keywords:** *HER2*, interstitial lung disease, non-small cell lung cancer, rechallenge, trastuzumab deruxtecan

## Abstract

**Introduction:**

Trastuzumab deruxtecan (T-DXd; DS-8201) has emerged as a standard later-line therapy for patients with HER2-mutant non-small cell lung cancer (NSCLC), but the optimal treatment strategy after disease progression using T-DXd remains unclear.

**Case presentation:**

We report the clinical course of a female lung adenocarcinoma patient with a HER2 exon 20 mutation who achieved long-term disease control with T-DXd despite multiple lines of chemotherapy, until interstitial pneumonia occurred when combined with a PD-1 inhibitor. After a two-month treatment break, the patient fully recovered from interstitial pneumonia using corticosteroid therapy. After recovery, two additional lines of chemotherapy were administered but produced limited benefit. Thus, T-DXd was reintroduced to control the worsening disease. Notably, T-DXd rechallenge, administered systemically along with local embolization, again provided clinical benefit despite prior radiographic progression on T-DXd and intervening systemic therapies. Overall, the patient achieved prolonged disease control during rechallenge (progression-free survival >14 months) without recurrence of interstitial lung disease (ILD) despite the extensive prior treatment exposure.

**Conclusion:**

This case differs from previously reported rechallenge experiences that mainly describe re-exposure after toxicity-related interruption as the rechallenge reported here followed documented disease progression and multiple intervening regimens, suggesting that, in carefully selected patients with HER2-mutant NSCLC who previously responded to T-DXd, rechallenge after progression could be explored as an option when therapeutic alternatives are limited. However, caution is advised and evidence from prospective and real-world cohorts is needed.

## Introduction

Human epidermal growth factor receptor 2 (*HER2*, also known as *ERBB2*) occurs in approximately 4% of non-small cell lung cancer (NSCLC) patients (1) and is predominantly mutually exclusive with other oncogenic drivers, with only a small proportion of *HER2*-mutant NSCLC patients harboring concurrent *EGFR* mutations, or *ALK* or *ROS1* rearrangement (2). Several antibody drug conjugates (ADCs) have been approved for the treatment of lung cancer (3). Trastuzumab deruxtecan (T-DXd; DS-8201), an anti-HER2 ADC, has shown promising antitumor activity in patients with pretreated, metastatic *HER2*-mutant NSCLC (4), including in Chinese patients (4). The drug was approved in October 2024 in China and is currently recommended as second- or later -line treatment for patients with pretreated metastatic *HER2*-mutated NSCLC (5). Its approval has expanded the therapeutic landscape for Chinese patients with advanced NSCLC who often demonstrate inadequate responses to first-line platinum–pemetrexed-based chemotherapy and have limited later-line treatment options (4, 6).

As T-DXd becomes increasingly used in routine clinical practice, an important and practical question has emerged regarding treatment sequencing after initial benefit: whether some selected patients might derive renewed clinical benefit from T-DXd re-administration following disease progression on prior T-DXd, particularly when intervening alternative systemic therapies has been administered. In real-world settings, many patients with advanced HER2-mutant NSCLC receive multiple subsequent regimens after progression, yet later-line efficacy is frequently modest, and clinicians are left with no other choice than to revisit previously active agents when alternative options are limited. Within this context, “rechallenge” is clinically relevant. Primary and acquired resistance to HER2−targeted ADCs such as trastuzumab emtansine (T-DM1) and T−DXd is increasingly understood to be multifactorial, involving alterations in HER2 expression, intracellular trafficking, and payload sensitivity (7, 8). Importantly, several resistance mechanisms appear to be dynamic and potentially reversible, providing a biological rationale for retreatment after an intervening therapeutic interval. At the same time, the safety profile of T-DXd remains central to treatment decision-making, with interstitial lung disease (ILD)/pneumonitis representing a well-recognized and potentially serious adverse event that can interrupt therapy and complicate subsequent management. In the DESTINY-Lung02 trial, adjudicated drug-related ILD/pneumonitis occurred in 12.9% of NSCLC patients treated with the recommended dose of T-DXd (5.4 mg/kg) (9). In DESTINY-Lung05, a trial of T-DXd in Chinese patients with *HER2*-mutant NSCLC with disease progression on or after at least one prior line of anticancer therapy, 7 of 72 patients (9.7%) developed any -grade ILD/pneumonitis (4). Beyond its direct clinical consequences, ILD/pneumonitis also poses diagnostic and therapeutic challenges in NSCLC, complicating the interpretation of radiologic findings when assessing antitumor efficacy and often necessitating interruption or cessation of ongoing systemic therapy (10). A pooled analysis of nine early-stage clinical trials of T-DXd, including 1,150 heavily pretreated patients, reported an overall incidence of 15.4% for adjudicated drug-related ILD/pneumonitis, with younger age (< 65 years) and T-DXd dose > 6.4 mg/kg among the risk factors identified (11). The recommended management comprises dose interruption, dose reduction or discontinuation of the antineoplastic agent, together with prompted administration of corticosteroids when clinically indicated (12); however, there is a lack of robust evidence to guide subsequent systemic treatment choices, particularly when considering whether and how to re-introduce T-DXd after progression or after clinically significant toxicity, and current reports are still largely anecdotal (11, 13).

Herein, we report the clinical course of a woman with *HER2*-mutant advanced NSCLC who received T-DXd in a later-line setting, and subsequently experienced disease progression on T-DXd, following which she was treated with other systemic treatments before being rechallenged with T-DXd in a subsequent line, achieving meaningful clinical benefit.

## Case presentation

A 53-year-old woman (body weight, 56 kg) presented with an unexplained cough lasting approximately one month in September 2019. Chest CT scan on September 25, 2019, revealed an occupying lesion in the upper lobe of the right lung, accompanied by mediastinal lymph node enlargement. CT-guided percutaneous biopsy confirmed infiltrating, moderately differentiated lung adenocarcinoma (acinar subtype). The tumor was negative for *ALK* rearrangement by Ventana ALK (D5F3) assay immunohistochemical assay, and PD−L1 expression was negative (TPS 0%, CPS <1). Next generation sequencing (NGS) performed on the initial lung biopsy specimen (September 26, 2019) identified a *HER2* exon 20 insertion mutation (p.Y772-A775dup; tissue abundance 17.11%), consistent with a diagnosis of HER2-mutant NSCLC; no other driver gene mutations, including *ALK*, *BRAF*, *EGFR*, *FGFR3*, *FGFR4*, *MET* and others were detected. The patient had an ECOG performance status of 1, with no history of smoking or alcohol use, no pulmonary comorbidities, and no other significant medical comorbidities. The patient was not receiving any concomitant medications at baseline.

**Figure 1:**
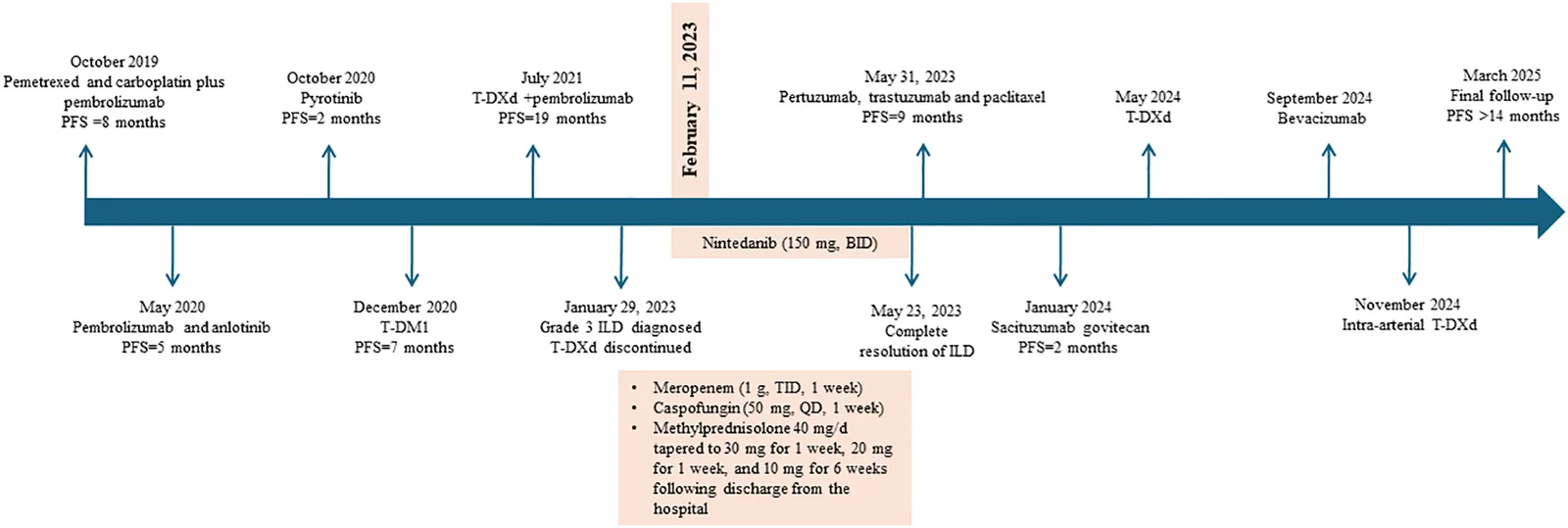
Treatment timeline of the 53-year-old woman with *HER2* mutant non-small cell lung cancer.

The timeline of treatments is shown in **Figure 1**. Starting from October 2019, the patient received first-line intravenous pemetrexed (500 mg/m²) and carboplatin (AUC = 4) combined with pembrolizumab (200 mg), administered intravenously on day 1 of each cycle for six cycles. She achieved partial response as the best overall response, and her clinical benefit corresponded to a progression-free survival (PFS) of approximately 8 months. In March 2020, maintenance therapy was initiated with pemetrexed (500 mg/m²) and pembrolizumab (200 mg) for three cycles, and bevacizumab (300 mg) was added on day 1 of the second maintenance cycle. During the first maintenance cycle, she developed worsening back pain, and a subsequent MRI suggested bone metastases and contrast-enhanced CT demonstrated enlargement of the primary tumor lesion in the upper lobe of the right lung; local radiotherapy was given as part of symptom-directed management. RECIST-defined progression was confirmed in May 2020, following which a change in systemic therapy was undertaken.

**Figure 2:**
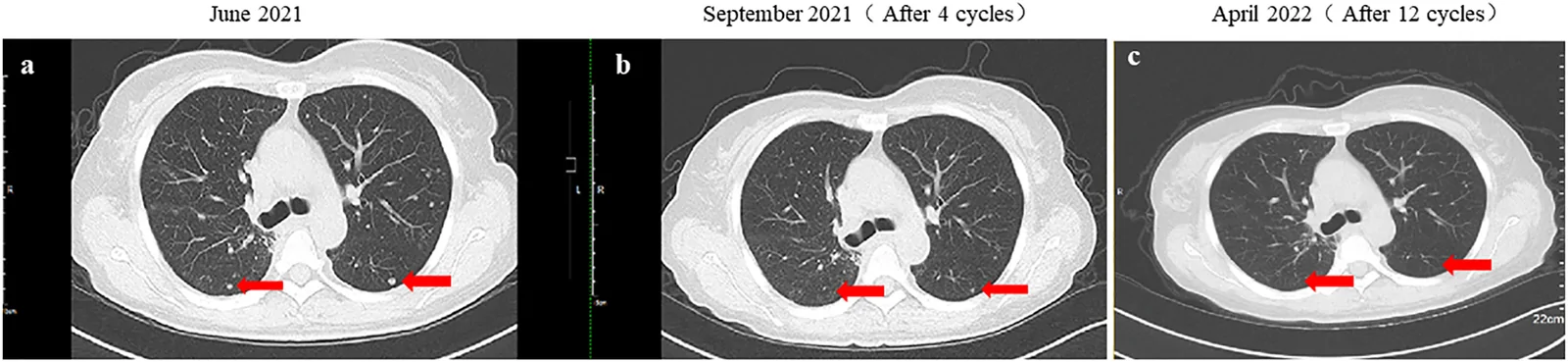
Chest CT scan showing **(a)** the baseline tumor lesion (arrows) before the 5^th^-line treatment with trastuzumab deruxtecan (T-DXd), **(b)** reductions in tumor lesion sizes (arrows) after 4 cycles of treatment with T-DXd, and **(c)** disappearance of tumor lesions after 12 cycles of treatment with T-DXd.

From May 2020, the patient received second-line pembrolizumab (200 mg, intravenous) combined with anlotinib (8 mg orally once daily, administered on a two-week-on/one-week-off schedule within 3-week cycles) for several months, and the clinical course reflected a PFS of approximately 5 months, after which imaging demonstrated progression (**Figure 2**). In October 2020, third-line pyrotinib, a small molecule TKI targeting HER2, was initiated at 240 mg/day; however, treatment was limited by tolerability, and the drug was discontinued due to gastrointestinal toxicities, including grade 2 diarrhea. The short duration of disease control on pyrotinib was consistent with a PFS of approximately 2 months, after which the patient proceeded to further HER2-directed therapy.

In December 2020, T-DM1 was prescribed as fourth-line treatment at a dose of 160/180 mg, given intravenously on day 1 of each cycle, for a total of eight cycles. The patient achieved a partial response as her best overall response, with a PFS of approximately 7 months. Nevertheless, by mid-2021, brain MRI scan suggested brain metastasis, while CT scan showed increased metastatic burden with progression in bilateral pulmonary lesions as well as osseous and extra-thoracic sites, including the sternum, multiple thoracic vertebrae, adnexa, and pelvis. CT-guided biopsy of the right ilium confirmed bone metastasis with histology showing metastatic moderately differentiated adenocarcinoma; however, the tissue obtained was insufficient for additional immunohistochemical staining, precluding HER protein re-assessment. NGS performed on this iliac biopsy specimen (June 28, 2021) showed an increased tissue abundance of *HER2* mutation (74.38%), supporting systemic progression and the need for subsequent-line therapy.

In July 2021, fifth line T-DXd was initiated at a dose of 3.6 mg/kg during the later course of therapy, achieving a partial response and a PFS of approximately 19 months (**Figure 2a**). During this period, serial assessments demonstrated meaningful anti-tumor activity and symptomatic stabilization, consistent with the patient’s prior pattern of response to HER2-directed therapy (**Figures 2b, c**). NGS on September 30, 2022, showed that *HER2* tissue abundance decreased 0.15%.

**Figure 3:**
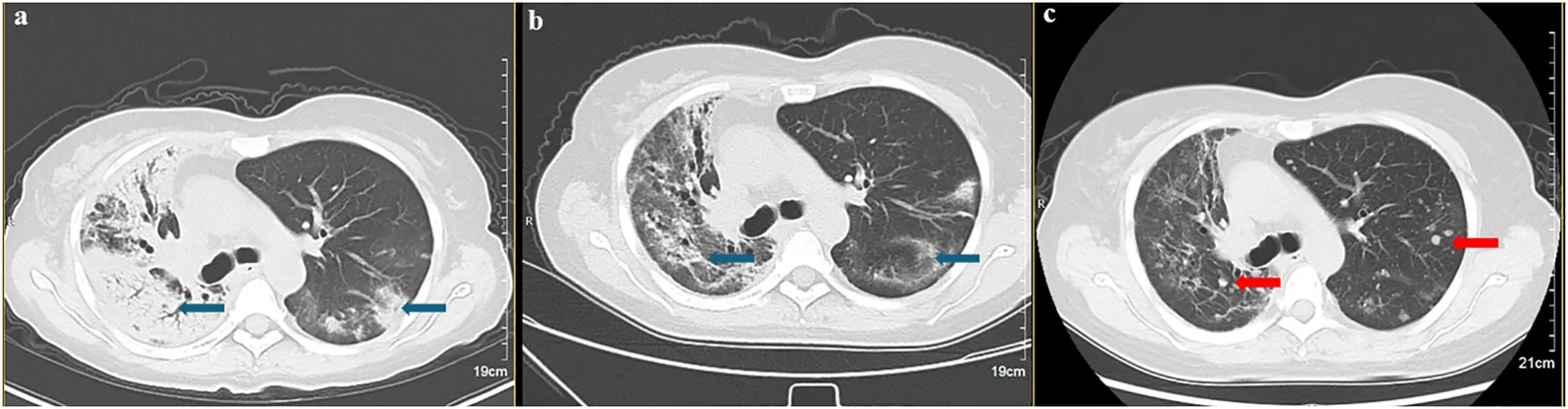
Chest CT scan showing cord-like interstitial thickening in the middle lobe (arrow), indicating interstitial lung disease (ILD) during the 5^th^-line treatment with T-DXd and pembrolizumab **(a)**. ILD was **(b)** partially resolved (arrow) 2 months after discontinuation of T-DXd, treatment with meropenem, methylprednisolone and nintedanib, and **(c)** ILD was completely resolved (arrow) 5 months after ILD treatment.

During the fifth-line T-DXd treatment period, NGS performed on peripheral blood (September 30, 2022) showed that *HER2* tissue abundance decreased to 0.15%. Toward the end of this interval, the patient developed features suggesting disease activity (**Figure 3a**), and her management course evolved accordingly. In October 2022, the re-examination of the lungs at another hospital indicated that the lesion showed a tendency to enlarge. In addition, T-DXd might be synergistic with an immune checkpoint inhibitor based on preclinical data and early reports of results in humans (14–16), we added pembrolizumab in combination with T-DXd in subsequent treatment of this patient.

On January 29, 2023, while receiving T-DXd and pembrolizumab, the patient developed fatigue, chest discomfort, and cough, and was diagnosed with grade 3 ILD. As it is recommended that T-DXd be permanently discontinued for grade 2 or higher ILD (17, 18), T-DXd was discontinued, and supportive management was initiated, which included intravenous meropenem (1 g three times daily) for 1 week, followed by caspofungin (50 mg once daily) for 1 week, together with methylprednisolone 40 mg/day that was tapered to 30 mg for 1 week, 20 mg for 1 week, and then 10 mg for 6 weeks following discharge. Out of concern over progression toward a fibrosing phenotype, nintedanib (150 mg twice daily) was initiated on February 11, 2023 to mitigate further fibroproliferative remodeling, and continued during recovery (**Figure 3b**). Chest CT on May 23, 2023, demonstrated complete radiologic resolution of ILD, and nintedanib was discontinued (**Figure 3c**). During this same post-T-DXd interval, systemic reassessment confirmed ongoing progressive disease, establishing that the patient had experienced progression on T-DXd and subsequently remained off T-DXd while alternative treatment strategies were pursued.

**Figure 4:**
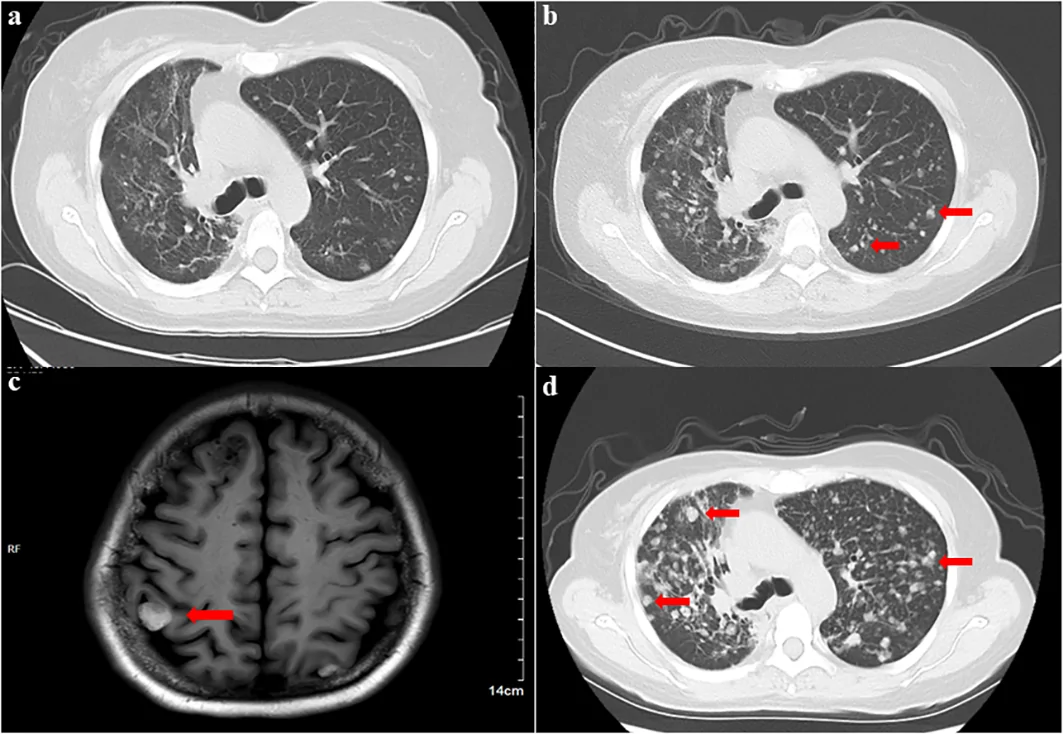
Chest CT scan showing **(a)** a reduction in the number and size of metastases in bilateral lungs with the 7^th^-line treatment with pertuzumab, trastuzumab, and albumin-bound paclitaxel. New metastatic lesions **(b)** in bilateral lungs can be seen in subsequent chest CT scans, and **(c)** cerebral MRI showing a new enhancement lesion (arrow). Chest CT scan at the final follow-up shows **(d)** an increase in the number and size of metastases in bilateral lungs (arrows).

The choice to pursue further HER2-targeted therapy was based on the patient’s documented prior response to T-DXd. On May 31, 2023, the patient initiated sixth-line therapy with pertuzumab (initial dose 840 mg, subsequent dose 420 mg, intravenous on day 0), trastuzumab (initial dose 8 mg/kg, subsequent dose 6 mg/kg, given intravenously on day 0), and albumin-bound paclitaxel (200 mg, given intravenously on day 1). Imaging demonstrated reduction in the number and size of metastatic lesions in bilateral lungs, and the duration of benefit on this regimen corresponded to a PFS of approximately 9 months (**Figure 4a**). Maintenance therapy was provided with intravenous pertuzumab (420 mg) and trastuzumab (330 mg) from November 2023 to January 2024 when imaging showed pulmonary progression (**Figure 4b**), and cerebral MRI showed new metastatic lesions (**Figure 4c**), confirming progressive disease.

In January 2024, the patient started seventh-line treatment with sacituzumab govitecan, a TROP2-targeting ADC (360 mg intravenously on days 1 and 8 of each 3-week cycle). The clinical course was complicated by treatment interruptions due to a traumatic bone fracture, and from March 2024 to May 2024, progression was confirmed, with a PFS of approximately 2 months. At this advanced stage, therapeutic options were limited, and the patient was no longer eligible for clinical trial enrollment due to her prior grade 3 pneumonitis/ILD. A careful risk–benefit assessment was performed involving a multidisciplinary team of thoracic oncology specialists at our center who reviewed available options with the patient, including standard chemotherapy and re-administration of T-DXd at a reduced dose with careful monitoring, recognizing both the prior activity of T-DXd and the need to distinguish the intent of re-treatment from an ILD-centered rechallenge narrative. Accordingly, in May 2024, T-DXd was reintroduced as eighth-line therapy, representing a rechallenge defined by re-administration of T-DXd after prior disease progression while receiving T-DXd, following an intervening interval of other systemic therapies (**Figure 1**). T-DXd was administered at a reduced dose (4.4 mg/kg) on a 3-week schedule. During rechallenge, the patient was monitored under an intensified safety protocol, including baseline and chest CT scan every 3–4 weeks, symptom screening at each visit, and immediate evaluation of any respiratory complaints. No recurrent ILD or pulmonary toxicity was observed. In September 2024, bevacizumab was added (300 mg on day 1 of each 3-week cycle). Throughout the rechallenge period, the patient did not develop symptoms or radiologic findings suggestive of recurrent ILD. In November 2024, due to worsening right leg pain and a large pelvic lesion burden, transarterial chemoembolization was undertaken, along with an intra-arterial T-DXd infusion (100 mg) *via* the right internal iliac artery (**Figure 1**). At the final follow-up in March 2025, chest CT demonstrated progression in bilateral pulmonary metastases (**Figure 4d**), but interestingly, the patient had remained progression-free for more than 10 months from the time of T-DXd rechallenge initiation. Her overall survival exceeded 71 months, with a prolonged disease course characterized by multiple lines of therapy, a clear interval of progression on T-DXd followed by intervening treatments, and subsequent meaningful disease control during a complex individualized treatment strategy with T-DXd rechallenge under close safety surveillance.

## Discussion

This case provides a clinically relevant observation of a complex individualized treatment strategy including successful T-DXd rechallenge in a heavily pretreated patient with HER2-mutant NSCLC who experienced disease progression on initial T-DXd, then received intervening alternative systemic therapies, and subsequently achieved renewed clinical benefit with a complex individualized treatment strategy, including successful T-DXd “rechallenge”, which is used to indicate re-treatment with T-DXd after documented progression on prior T-DXd, following intervening anticancer treatments, which distinguishes this strategy from treatment beyond progression and also from rechallenge defined purely by interruption for drug-related toxicity.

The clinical activity of T-DXd in previously treated HER2-mutant metastatic NSCLC has been established in DESTINY-Lung01 and 02 trials (9, 19), where confirmed objective response rates were approximately 49% with T-DXd 5.4 mg/kg and 56% with T-DXd 6.4 mg/kg, with durable responses and a safety profile that supported adoption of T-DXd 5.4 mg/kg as the standard dose in this setting. Subsequent updates with longer follow-up and patient-reported outcomes reinforced the consistency of clinical benefit and the overall tolerability of T-DXd at the approved dose (10). In Chinese patients, the DESTINY-Lung05 trial similarly reported clinically meaningful activity, supporting the broader implementation of T-DXd in routine practice in China (4).

Given the well−recognized risk of immune−related pneumonitis with PD−1 inhibitors including pembrolizumab (20, 21), and an increased risk of ILD in patients receiving T−DXd (22, 23), the cause of ILD in our patient cannot be clearly attributed to pembrolizumab or T−DXd, or the joint effect of the two agents. What makes this case distinct is that renewed disease control was achieved when T-DXd was restarted as a part of a complex individualized treatment strategy after documented radiographic progression and intervening therapy with re-exposure to T-DXd, rather than simply resuming treatment after a brief interruption for management of adverse events. In contrast, most published “rechallenge” reports describe re-treatment once ILD/pneumonitis has resolved, including successful cases after grade 3 ILD in HER2-mutant NSCLC and after lower-grade ILD events in other HER2-driven tumors (11). In addition, a pooled analysis evaluating T-DXd retreatment after recovery from grade 1 ILD suggested that re-exposure is feasible in carefully selected patients, although ILD recurrence occurred in about one-third of retreated patients and requires structured monitoring and guideline-based management (12). However, these ILD-centered scenarios largely represent temporary interruption followed by resumption of the same line of therapy, whereas our case is closer to a sequencing question: can T-DXd recapture benefit after prior T-DXd progression when later-line options are limited?

The biological rationale for post-progression T-DXd rechallenge remains uncertain, though several clinically plausible considerations apply including mechanisms of resistance to HER2−directed ADCs. HER2 downregulation or heterogeneity may be reversible after cessation of HER2−targeted therapy or following cytotoxic regimens that eliminate HER2−low subclones. Impaired internalization or lysosomal trafficking can also potentially be restored after intervening therapies that modify tumor cell biology or the tumor microenvironment, and payload−specific resistance. For instance, intervening therapies may shift clonal composition, alter tumor microenvironment pressures, or reduce the dominance of resistant subclones, thereby allowing re-emergence of T-DXd-sensitive disease and creating a window of renewed sensitivity to T−DXd. In our present case, we believe that the intervening regimen incorporating pertuzumab plus trastuzumab may have contributed to disease control during the interval, despite available data suggesting dual anti-HER2 strategies have only modest activity in HER2-altered NSCLC, emphasizing that the subsequent duration of benefit after T-DXd re-administration is unlikely to be explained by anti-HER2 antibodies alone (13). Mechanistically, resistance to T-DXd has been linked to factors such as reduced HER2 expression, drug efflux, and downstream signaling alterations, and it remains unknown whether a drug-free interval and the duration required for such an interval can reverse these resistance mechanisms, or whether spatial heterogeneity enables re-selection of sensitive clones over time. Real-world evidence continues to support the effectiveness of T-DXd in diverse practice settings, but specific evidence for rechallenge after progression is still limited. Importantly, the ILD history in this patient remains clinically essential because it influenced eligibility for subsequent trials and required increased vigilance during rechallenge, consistent with emerging experience that dose-modified re-exposure can be considered in selected circumstances, although our case differs by being a post-progression rechallenge rather than a toxicity-driven resumption. Although clinical evidence remains limited, this biological framework supports the plausibility of benefit in carefully selected patients, as illustrated in our case. These additions provide a clearer mechanistic rationale and to situate our case within the evolving understanding of ADC resistance and re−sensitization. However, the current case received multiple lines of treatment, concomitant therapies and local treatments, which may introduce bias and complicate the interpretation of treatment outcomes.

Several treatment decisions in this case were off−label and highly individualized, including the use of T−DXd plus pembrolizumab, T−DXd rechallenge after grade 3 ILD, addition of bevacizumab, and intra−arterial administration of T−DXd. All of these approaches were undertaken outside of established standard−of−care frameworks, with each decision being made through multidisciplinary discussion and shared decision−making with the patient, based on limited remaining treatment options and careful evaluation of potential risks and benefits. These strategies are presented as individualized clinical choices, not as recommended or validated therapeutic approaches. Regarding the addition of pembrolizumab to T−DXd, the combination was used in an exploratory context as there is limited clinical evidence supporting this regimen and the potential for increased ILD risk when combining an ADC with immunotherapy. In the rapidly advancing treatment landscape for HER2−mutant NSCLC, several emerging HER2−directed TKIs provide important therapeutic alternatives for patients who cannot safely continue or resume T−DXd, particularly after ILD. Recent data demonstrate that zongertinib achieves clinically meaningful responses in previously treated HER2−mutant disease, supporting its role as a next−line targeted option (24). Similarly, the SOHO−01 study reported highlights the activity of sevabertinib (25), further expanding the spectrum of HER2−TKIs under investigation. Importantly, early real−world evidence suggests that zongertinib may be feasible even in patients with prior T−DXd–associated ILD (26), a population in whom ADC rechallenge carries substantial risk. Together, these findings underscore the growing availability of HER2−targeted strategies and contextualize the individualized treatment decisions in our case within a broader set of evolving therapeutic options.

We acknowledge several limitations. The molecular testing was performed on different specimen types (tissue and blood) over time, and HER2 protein expression could not be reassessed at progression due to insufficient tissue from the iliac biopsy. In addition, the current case received multiple lines of treatment, concomitant therapies, and local treatments, which may introduce bias and complicate the interpretation of treatment outcomes.

In conclusion, this case report suggests that, when alternatives are exhausted, T-DXd rechallenge as part of a complex individualized treatment strategy after progression following an intervening treatment interval may be considered in carefully selected HER2-mutant NSCLC patients. However, caution is advised and evidence from prospective and real-world cohorts is needed.

## Patient perspective

From the patient’s perspective, limited treatment options necessitated the decision to receive T-DXd after multiple prior therapies, including progression on T-DXd, offering a potential opportunity for disease control. Prior ILD required careful monitoring and dose adjustments, with trust in the multidisciplinary team and clear communication about risks and benefits guiding the decision.

## Data Availability

The original contributions presented in the study are included in the article/supplementary material. Further inquiries can be directed to the corresponding authors.
